# Sutureless Technique for Primary Total Anomalous Pulmonary Venous Connection Repair: An Updated Meta-Analysis

**DOI:** 10.3389/fcvm.2022.890575

**Published:** 2022-04-28

**Authors:** Lu Zhao, Zhengxia Pan, Chun Wu, Lianju Shen, Yuhao Wu

**Affiliations:** ^1^Department of Cardiothoracic Surgery, Children’s Hospital of Chongqing Medical University, Chongqing, China; ^2^Chongqing Key Laboratory of Pediatrics, Ministry of Education Key Laboratory of Child Development and Disorders, China International Science and Technology Cooperation Base of Child Development and Critical Disorders, National Clinical Research Center for Child Health and Disorders, Chongqing, China

**Keywords:** total anomalous pulmonary venous connection, sutureless technique, congenital heart disease, children, surgery

## Abstract

**Background:**

An updated meta-analysis was performed to explore the clinical outcomes following the sutureless repair in patients with total anomalous pulmonary venous connection (TAPVC) and compare outcomes between the sutureless technique and conventional surgery.

**Methods:**

A systematic search of PubMed, Ovid-Embase, and Cochrane Library (CENTRAL) for relevant published studies that reported the clinical outcomes of the sutureless technique in children with TAPVC was performed in February 2022. The publication language was restricted to English.

**Results:**

Eleven studies were included involving 771 patients in total. Following the sutureless technique, the incidences of postoperative pulmonary venous obstruction (PVO) and reoperations due to PVO were 3.3% [95% confidence interval (CI), 1.3–5.3%] and 1.8% (95% CI, 0.3–3.3%), respectively. The early and late mortality rates were 3.2% (95% CI, 1.0–5.3%) and 2.5% (95% CI, 0.7–4.3%), respectively. Compared with conventional surgery, the sutureless technique significantly reduced the incidences of postoperative PVO [odds ratio (OR), 0.16; 95% CI, 0.08–0.33; *P* < 0.00001], reoperations due to PVO (OR, 0.25; 95% CI, 0.10–0.63; *P* = 0.003), and early mortality (OR, 0.40; 95% CI, 0.21–0.79; *P* = 0.008). However, no significant difference was found between conventional surgery and the sutureless technique concerning late mortality (OR, 0.63; 95% CI, 0.13–3.00; *P* = 0.58).

**Conclusion:**

The sutureless technique is superior to conventional surgery for the primary repair of TAPVC concerning postoperative PVO, reoperations due to PVO, and early mortality. However, the level of evidence is of low quality. Prospective cohort studies or randomized control trials (RCTs) should be performed to evaluate the effectiveness of sutureless techniques for primary TAPVC repair.

## Introduction

Total anomalous pulmonary venous connection (TAPVC) is a rare congenital heart malformation, accounting for nearly 1% of all congenital heart diseases (CHDs) (1). In patients with TAPVC, the pulmonary veins (PVs) are connected to the systemic venous system. TAPVC can occur in isolation or in combination with other malformations such as right atrial isomerism (RAI) and single ventricle (SV) (2, 3). If left untreated, TAPVC leads to a mortality of up to 80% in the first year of life (4). Because of advances in surgical techniques and perioperative management, mortality is dramatically decreased (5). Using conventional surgery (6), a side-to-side anastomosis is achieved between the left atrium (LA) and pulmonary venous confluence (PVC). However, surgical trauma to the intima of PVs is significant using conventional surgery, and postoperative pulmonary venous obstruction (PVO), which is associated with increased mortality (7), remains a challenge for pediatric cardiac surgeons.

**FIGURE 1:**
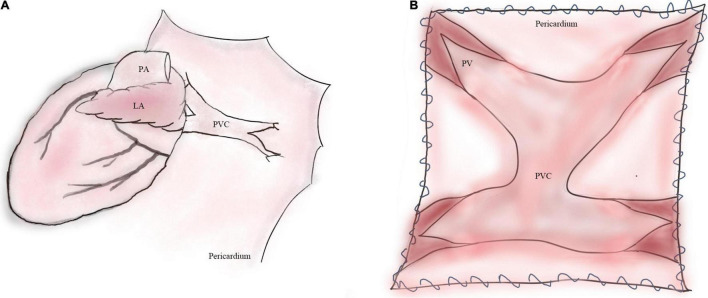
Operative illustrations of primary sutureless repair for TAPVC. **(A)** Illustration of TAPVC. **(B)** The left atrial wall is anastomosed to the posterior pericardium. LA, left atrium; PA, pulmonary artery; PVC, pulmonary venous confluence; PV, pulmonary vein.

The sutureless technique is primarily created to relieve secondary PVO after initial TAPVC surgery. Using the sutureless technique, a neo-LA is created by anastomosing the LA to pericardium *in situ* (Figure 1). The sutureless technique not only avoids mechanical stimulus to PVs but also minimizes the distortion of the suture line (8). In recent years, to prevent potentially postoperative PVO, the sutureless technique, as a prophylactic method, has been adopted for the primary repair of TAPVC (1, 5, 6). However, based on current evidence, the outcomes of the sutureless technique are not completely promising (9, 10).

In 2018, Wu et al. (6) published a meta-analysis comparing the sutureless technique with conventional surgery for TAPVC. They have found that the sutureless technique can reduce postoperative PVO and reoperations due to PVO. However, several two-arm studies comparing the sutureless technique versus conventional repair have been subsequently published. At the same time, the current evidence regarding the outcomes of the sutureless technique versus conventional surgery remains inconclusive. Therefore, the present study aimed to synthesize the evidence with respect to clinical outcomes following primary sutureless repair.

## Materials and Methods

The protocol of this study was registered on PROSPERO website (CRD42022312156). In this study, we aimed to perform an updated meta-analysis and identify potential factors that affected the clinical outcomes following primary sutureless repair in patients with TAPVC. Our study was performed following the updated Preferred Reporting Items for Systematic Reviews and Meta-Analyses (PRISMA) reporting guidelines (11).

### Literature Search

In February 2022, a systematic search of the Ovid-Embase, PubMed, and the Cochrane Library (CENTRAL) for the relevant published studies that reported the outcomes of the sutureless technique in children with TAPVC was performed. The publication language was restricted to English. The key search strategy is displayed as follows:

#1 Total anomalous pulmonary venous connection OR total anomalous pulmonary venous drainage
#2 Sutureless technique OR sutureless surgery OR sutureless repair
#3 #1 AND #2

For additional eligible studies, we manually reviewed references from our included studies and other relevant literature. Two reviewers (LZ and YW) independently searched all the publications in the literature.

### Study Selection

The specific framework in terms of Population, Intervention, Comparator, Outcome, and Study Design (PICOS) was constructed as follows: (P) patients with untreated TAPVC; (I) the sutureless technique; (C) conventional surgery; (O) postoperative PVO, reoperations due to PVO, and early and mortality; and (S) case series, cohort or case-controlled studies.

We included a study in this meta-analysis when any one of the following criteria was met: (1) single-arm case series reporting clinical outcomes regarding the primary use of sutureless technique for patients with TAPVC; (2) two-arm case-controlled studies, cohort studies, or randomized controlled trials (RCTs) comparing the sutureless technique with conventional surgery for TAPVC.

We excluded a study from this meta-analysis when any one of the following criteria was met: (1) multiple studies were based on the same population and study period; (2) the sample size of the involved patients was fewer than three cases; (3) studies reported the secondary use of sutureless technique for postoperative PVO; (4) studies reported the use of sutureless technique for congenital pulmonary venous stenosis; (5) outcomes (i.e., postoperative PVO, mortality, and reoperations) regarding sutureless technique for TAPVC were not reported. When several studies were based on the same patients and study period, the study with the most complete dataset was included. Reviews, editorial materials, and conference abstracts were also excluded. Two reviewers (CW and YW) independently screened all the publications included in this study. With the assistance of a third reviewer (ZP), disagreements on the eligibility of the published studies were resolved.

### Data Extraction

The data were extracted by both reviewers (LZ and YW) independently, and disagreement was resolved with the assistance of a third reviewer (LS). The following data were extracted: (1) baseline characteristics of included studies: first author, year of publication, study area, types of study design, sample size of the included population, category of TAPVC, surgical approach, operative age and weight, preoperative PVO, cardiopulmonary bypass (CPB) duration, aortic cross clamp (ACC) time, and the follow-up period; (2) primary outcome: postoperative PVO; (3) secondary outcomes: reoperations due to PVO and early and late mortality.

### Definition of Variables

Early mortality was defined as the postoperative death before discharge or within a month after surgery. Late death was defined as death after discharge. Postoperative PVO was defined as anastomotic stricture or peripheral stenosis of individual PVs after primary surgery. Reoperations due to PVO referred to operations including surgery and interventional treatment performed for PVO.

### Quality Assessment and Risk of Bias

Quality assessment was performed by two reviewers (LZ and YW) independently. Any discrepancies in the assessed quality were resolved by a third reviewer (LS) by consensus. The risk of bias (RoB) for the single-arm case series was evaluated using the methodological index for non-randomized studies (MINORS) guidelines (12). The RoB of case-controlled studies was evaluated using the risk of bias in non-randomized studies of interventions (ROBINS-I) tool (13). Each study was assessed in seven domains, and signaling questions were framed to evaluate RoB within each domain.

### Statistical Analysis

Statistical analyses were performed using *Stata 12.0*, *Revman 5.3*, and *OpenMetaAnalyst* software. The χ^2^-Q statistics and the *I*^2^ statistics were used to assess heterogeneity, with *I*^2^ > 50% indicating significant heterogeneity.

To investigate the clinical outcomes of the sutureless technique, we mainly included and analyzed single-arm case series in this setting. We performed meta-analyses of the prevalence (early mortality, late mortality, postoperative PVO, and reoperations due to PVO) using the inverse variance method of DerSimonian–Laird (D + L Method). We adopted random-effect models because of the likelihood of the inter-study heterogeneity of single-arm case series.

To compare the clinical outcomes of the sutureless technique versus conventional surgery, we included two-arm case-controlled studies in this setting. The odds ratio (OR) was adopted for dichotomous data. If the *I*^2^ statistic was greater than 50%, a randomized-effect model was adopted; otherwise, a fixed-effect model was adopted for data synthesis.

To find the sources of heterogeneity, meta-regression and subgroup analyses were used. Univariate meta-regression analysis was performed using random models with continuous covariates including operative age and weight, CPB duration, ACC time, preoperative PVO, and infracardiac or mixed TAPVC percentages. Subgroup analysis was conducted based on dichotomous covariates including operative age, study area, year of publication and percentages of preoperative PVO, and infracardiac or mixed TAPVC. *P*-values for interaction were calculated to compare differences between subgroups. Funnel plots were produced to assess the publication bias. Sensitivity analysis was performed to assess the robustness of our synthesized results. Sensitivity analysis was performed using the leave-one-out method. A *P*-value < 0.05 was considered statistically significant.

## Results

### Characteristics of the Included Studies and Quality Assessment

**FIGURE 2:**
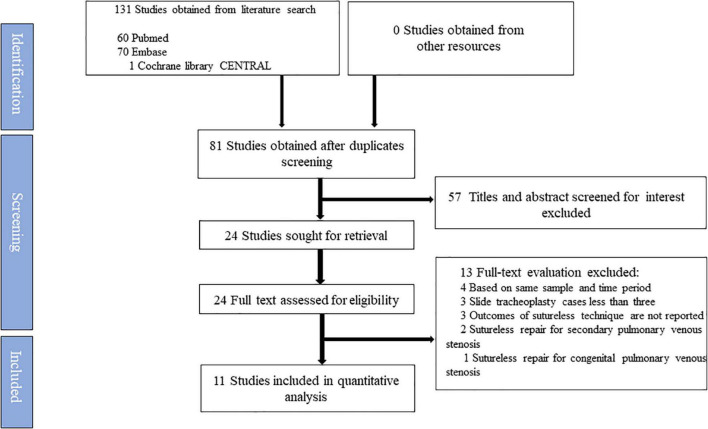
Flow diagram according to the updated Preferred Reporting Items for Systematic Review and Meta-Analysis (PRISMA) protocol recommendations.

A total of 131 studies were obtained from the electronic databases initially. However, no additional studies were identified manually on the reference lists of the retained studies. Twenty-four studies were deemed qualified for the full-text assessment, of which 13 studies were excluded (Supplementary Table 1). Eventually, this systematic review was based on 11 studies (8, 14–23). The flowchart depicting the search strategy is shown in Figure 2.

Four studies were single-arm case series (14–17) that reported the clinical outcomes of the sutureless technique for children with TAPVC (Table 1). Seven studies were case-controlled studies (8, 18–23) comparing the sutureless technique with conventional surgery, and no cohort study or RCT was identified based on our literature search. Finally, 771 patients were included in this study, of whom 315 patients were in the sutureless technique group and 456 patients in conventional surgery group. All the included studies were used for quantitative analysis. Of the 11 studies, 3 studies originated from North America or Europe, and the remaining studies were from Asia.

**TABLE 1:** Baseline characteristics of the included studies.

First author, year of publication, study area	Study design	Operative approaches	Study size (*n*)	Category of TAPVC (*n*)	Mean or median operative age (days)	Preoperative PVO (*n*)	Follow-up period (months)
Yoshimura, 2010, Japan (14)	Case series	Sutureless technique	3	Supracardiac 1 Infracardiac 2	12.7	3	15
Azakie, 2011, United States (15)	Case series	Sutureless technique	18	Supracardiac 10 Infracardiac 5 Cardiac 1 Mixed 2	18	14	34
Mueller, 2013, Switzerland (16)	Case series	Sutureless technique	7	Supracardiac 4 Infracardiac 3	6.4	3	54
Jung, 2016, Korea (17)	Case series	Sutureless technique	21	Supracardiac 9 Infracardiac 10 Mixed 2	21	13	3.6
Lo Rito, 2015, Canada (18)	Case-controlled studies	Sutureless technique	69	Supracardiac 32 Infracardiac 16 Cardiac 6 Mixed 15	18	33	76.8
		Conventional surgery	126	Supracardiac 59 Infracardiac 20 Cardiac 33 Mixed 14	36	44	
Zhang, 2016, China (19)	Case-controlled studies	Sutureless technique	70	Supracardiac 57 Infracardiac 9 Mixed 4	68	19	12
		Conventional surgery	70	Supracardiac 58 Infracardiac 7 Mixed 5	55	19	
Zhu, 2019, China (20)	Case-controlled studies	Sutureless technique	20	Supracardiac	198	NA	36
		Conventional surgery	23	Supracardiac	202		
Shi, 2021, China (8)	Case-controlled studies	Sutureless technique	15	Infracardiac	17	9	16
		Conventional surgery	67	Infracardiac	27	36	35
Liufu, 2021, China (21)	Case-controlled studies	Sutureless technique	36	Infracardiac	21	21	12
		Conventional surgery	27	Infracardiac	15	13	
Qiu, 2021, China (22)	Case-controlled studies	Sutureless technique	43	Supracardiac	60	13	65
		Conventional surgery	130	Supracardiac	90	39	
Xia, 2021, China (23)	Case-controlled studies	Sutureless technique	13	NA	90	13	85
		Conventional surgery	13		90	13	

*TAPVC, total anomalous pulmonary venous connection NA, not available.*

For single-arm studies (14–17), we assessed RoB in eight items using the MINORS guideline. The scoring approaches are described in Supplementary Table 2. Two studies were deemed moderate quality (14, 17). The remaining two studies were deemed high quality (15, 16). For two-arm case controlled studies, we assessed RoB in seven domains using the ROBINS-I tool. The scoring approaches are described in Supplementary Table 3. Only two studies used propensity score matching to adjust for confounders and avoid selection bias (19, 23). These two studies were deemed high quality (19, 23). The remaining five studies were deemed moderate quality (8, 18, 20–22).

### Outcomes of the Sutureless Technique Based on Single-Arm Analysis

#### Primary Outcome: Postoperative Pulmonary Venous Obstruction

**FIGURE 3:**
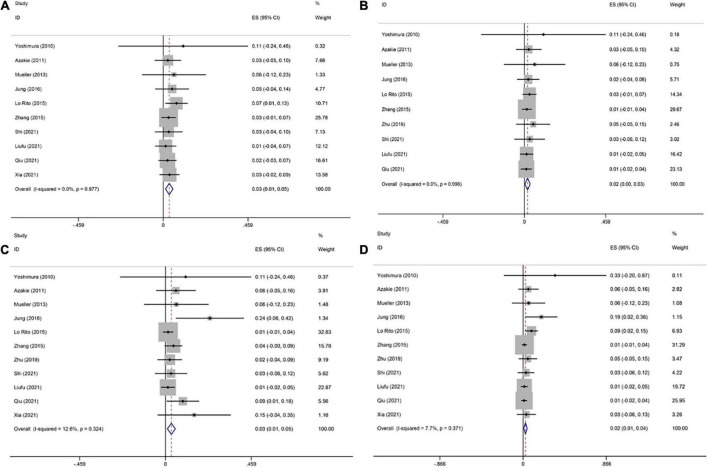
Forest plots of postoperative pulmonary venous obstruction (PVO), early and late mortality, and reoperations due to PVO after sutureless technique. **(A)** Forest plot of incidence of postoperative PVO. **(B)** Forest plot of reoperations due to PVO. **(C)** Forest plot of early mortality. **(D)** Forest plot of late mortality.

One study (20) did not report postoperative PVO following the sutureless repair, therefore this meta-analysis was based on 10 studies (8, 14–19, 21–23). The pooled estimate of postoperative PVO was 3.3% [95% confidence interval (CI), 1.3–5.3%; *I*^2^ = 0; Figure 3A] following the sutureless repair.

#### Secondary Outcome: Reoperations Due to Pulmonary Venous Obstruction

One study (23) did not report reoperations due to PVO following the sutureless repair, therefore this meta-analysis was based on 10 studies (8, 14–22). The pooled estimate of postoperative PVO was 1.8% (95% CI, 0.3–3.3%; *I*^2^ = 0; Figure 3B) following the sutureless repair.

#### Secondary Outcome: Early Death

This meta-analysis was based on 11 studies (8, 14–23). The pooled estimate of early death was 3.2% (95% CI, 1.0–5.3%, *I*^2^ = 12.6%, Figure 3C) following the sutureless repair.

#### Secondary Outcome: Late Death

This meta-analysis was based on 11 studies (8, 14–23). The pooled estimate of late death was 2.5% (95% CI, 0.7–4.3%; *I*^2^ = 7.7%; Figure 3D) following the sutureless repair.

#### Subgroup Analysis

Subgroup analysis of the primary outcome was conducted based on the operative age, study area, year of publication and percentages of preoperative PVO, and infracardiac or mixed TAPVC (Table 2). However, in the subgroup analysis of postoperative PVO, no significant differences were found between different subgroups.

**TABLE 2:** Subgroup analysis of primary outcomes in patients with TAPVC following sutureless repair based on single-arm analysis.

Primary outcomes	Subgroups	Studies (*n*)	Pooled estimate (95% CI)	Heterogeneity (*I*^2^)	*P*-value	*P* for interaction
Postoperative PVO	Average or median age					
	<30 days	7	3.8% (0.8–6.8%)	0	0.013	0.64
	≥30 days	3	2.8% (0.2–5.5%)	0	0.037	
	Study area					
	Asia	7	2.8% (0.5–5.0%)	0	0.015	0.33
	North America or Europe	3	5.3% (0.8–9.8%)	0	0.021	
	Year of publication*					
	Before 2016	5	4.0% (0.1–6.9%)	0	0.008	0.52
	After 2016	5	2.7% (0–5.4%)	0	0.054	
	Preoperative PVO (%)					
	≥50%	7	3.7% (0.1–6.3%)	0	0.007	0.66
	<50%	3	2.8% (−0.3–5.8%)	0	0.073	
	Infracardiac or mixed TAPVC (%)					
	≥50%	5	2.9% (−0.3–6.1%)	0	0.08	0.77
	<50%	5	3.5% (0.1–6.0%)	0	0.007	

*PVO, pulmonary venous obstruction.*

*We compared pooling results of the last 5 years to those of 5 years ago.

#### Meta-Regression Analysis

Meta-regression analysis of the primary outcome based on single-arm analysis was conducted based on the operative age and weight, percentage of preoperative PVO, and infracardiac or mixed TAPVC, CPB duration, and ACC time (Supplementary Table 4). In the univariate meta-regression analysis of postoperative PVO, no significant risk factors for postoperative PVO were identified. Therefore, the multivariate meta-regression analysis was not performed.

#### Sensitivity Analysis

To assess the robustness of our synthesized results, we performed sensitivity analyses. We performed a sensitivity analysis using the leave-one-out method. The overall estimates were robust and not overinfluenced by any of the included studies (Supplementary Figure 1).

### The Sutureless Technique Versus Conventional Surgery Based on Two-Arm Studies

#### Primary Outcome: Postoperative Pulmonary Venous Obstruction

**FIGURE 4:**
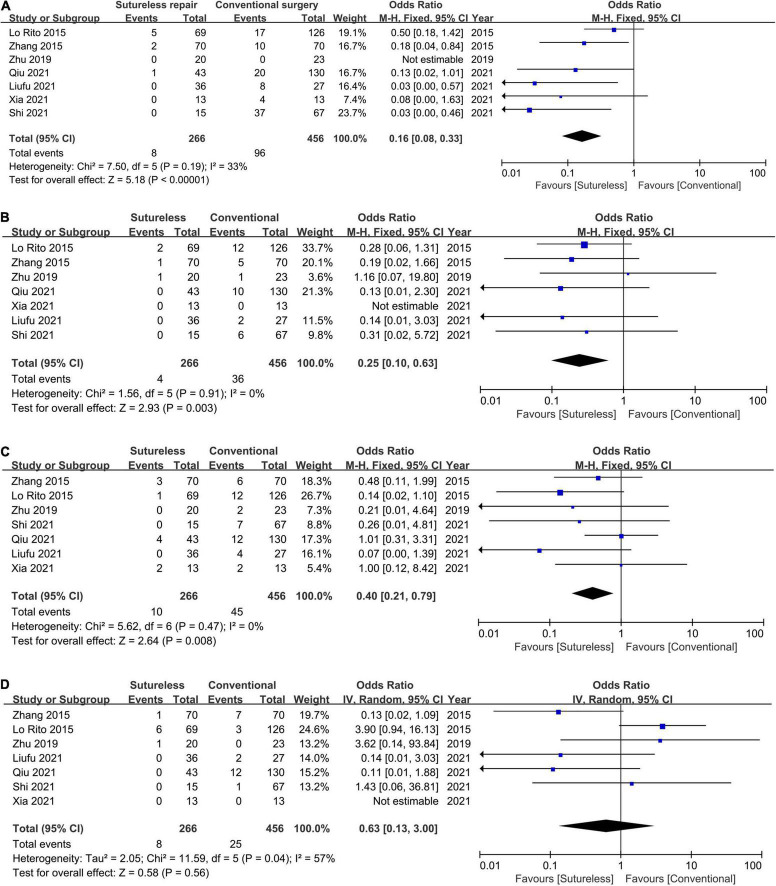
Forest plot of odds ratio (OR) of post-operative PVO, early and late mortality, and reoperations due to PVO. **(A)** Forest plot of OR of postoperative PVO and pooled estimate OR of 0.16 in favor of the sutureless technique. **(B)** Forest plot of OR of reoperations due to PVO and pooled estimate OR of 0.25 in favor of the sutureless technique. **(C)** Forest plot of OR of early mortality and pooled estimate OR of 0.40 in favor of the sutureless technique. **(D)** Forest plot of OR of late mortality and no statistical difference was found.

A total of six studies (8, 18, 19, 21–23) compared the incidence of postoperative PVO after repair using both procedures, and they were involved in this meta-analysis. The meta-analysis indicated that the sutureless technique was associated with a lower incidence of postoperative PVO than conventional surgery (OR, 0.16; 95% CI, 0.08–0.33; *P* < 0.00001; *I*^2^ = 33%; Figure 4A). Because of the limited number of included studies, funnel plot was not produced.

#### Secondary Outcome: Reoperations Due to Pulmonary Venous Obstruction

A total of six studies (8, 18–22) compared the incidence of reoperations due to PVO, and they were involved in this meta-analysis. The meta-analysis suggested that the sutureless technique was associated with a lower incidence of reoperations due to PVO (OR, 0.25; 95% CI, 0.10–0.63; *P* = 0.003; *I*^2^ = 0; Figure 4B).

#### Secondary Outcome: Early Death

A total of seven studies (8, 18–23) compared the early mortality after repair using both procedures, and they were involved in this meta-analysis. The meta-analysis showed that the sutureless technique was associated with a lower early mortality than conventional surgery (OR, 0.40; 95% CI, 0.21–0.79; *P* = 0.008; *I*^2^ = 0, Figure 4C).

#### Secondary Outcome: Late Death

A total of six studies (8, 18–22) compared the late mortality after repair of both procedures, and they were involved in this meta-analysis. This meta-analysis revealed that the late mortality was not significantly different between the sutureless technique and conventional surgery (OR, 0.63; 95% CI, 0.13–3.00; *P* = 0.58; *I*^2^ = 57%; Figure 4D).

#### Subgroup Analysis

Because only one two-arm study was originated from the North America (18), subgroup analysis based on the study area was not performed. Therefore, subgroup analysis of the primary outcome was conducted based only on the operative age, year of publication and percentage of preoperative PVO, and infracardiac or mixed TAPVC (Table 3). We found that compared with studies published earlier than 2016, the sutureless technique significantly reduced risks for postoperative PVO in studies published during the last 5 years (*P* for interaction = 0.03).

**TABLE 3:** Subgroup analysis of postoperative PVO based on two-arm studies*.

Primary outcomes	Subgroups	Studies (*n*)	OR (95% CI)	Heterogeneity (*I*^2^)	*P*-value	*P* for interaction
Postoperative PVO	Average or median age					
	<30 days	3	0.18 (0.08–0.42)	70.6%	<0.0001	0.72
	≥30 days	3	0.14 (0.04–0.44)	0	0.001	
	Year of publication^&^					
	Before 2016	2	0.35 (0.15–0.82)	16.4%	0.016	0.03
	After 2016	4	0.06 (0.02–0.22)	0	<0.0001	
	Preoperative PVO (%)					
	≥30%	2	0.04 (0.01–0.30)	0	0.002	0.12
	<30%	4	0.22 (0.11–0.46)	32.2%	<0.0001	
	Infracardiac or mixed TAPVC (%)					
	≥50%	2	0.03 (0.01–0.22)	0	0.001	0.08
	<50%	3	0.28 (0.13–0.61)	3%	0.001	

*Among included six two-arm studies, only one study was from United States, so the subgroup analysis based on study area was not performed.

*PVO, pulmonary venous obstruction.*

*^&^We compared pooling results of the last 5 years to those of 5 years ago.*

#### Meta-Regression Analysis

Meta-regression analysis of the primary outcome based on two-arm studies was conducted based on the operative age and weight, percentages of preoperative PVO, and infracardiac or mixed TAPVC, CPB duration, and ACC time (Supplementary Table 5). In the univariate meta-regression analysis of postoperative PVO, no significant risk factors for postoperative PVO were identified. Thus, the multivariate meta-regression analysis was not performed.

#### Sensitivity Analysis

We performed a sensitivity analysis using the leave-one-out method. The overall OR of early mortality changed significantly after excluding one study performed by Lo Rito et al. (18). Compared with conventional surgery, this study reported a very low early mortality following the sutureless repair. The overall ORs of the other three outcomes were robust and not overinfluenced by any of the included studies (Supplementary Figure 2).

## Discussion

Postoperative PVO continues to be the most significant complication in patients following TAPVC repair. For the last two decades, technical modifications for TAPVC repair have been focused on reducing the incidence of postoperative PVO (18, 24). The rationale underlying primary sutureless repair is to avoid the direct mechanical stimulus to the PVs, and the main disadvantage of the sutureless technique is potential bleeding from the gap between the pericardium and the PVC into the posterior mediastinum. However, only one actual patient (14) had with massive bleeding in our included studies. In the present study, we mainly found that based on the latest evidence, the incidence of postoperative PVO was 3.3% following primary sutureless repair in patients with TAPVC. At the same time, after primary sutureless repair, only 1.8% of included patients required reoperations due to PVO. Early and late mortality rates were 3.2 and 2.5%, respectively. Furthermore, we also compared the sutureless technique with conventional surgery. We observed that compared with conventional surgery, the sutureless technique was associated with lower incidences of postoperative PVO and reoperations due to PVO. The sutureless technique also effectively reduced early mortality. However, the sutureless technique was not superior to conventional surgery regarding long-term survival.

Using subgroup and meta-regression analyses, we aimed to identify the sources of heterogeneity and potential factors for postoperative PVO. However, based on single-arm studies, subgroup analysis (Table 2) and meta-regression analysis (Supplementary Table 4) did not identify any significant factors for postoperative PVO. Based on two-arm studies, compared with studies published earlier than 2016, the sutureless technique significantly reduced risks for postoperative PVO in studies published during the last 5 years (Table 3). In the literature, concerning postoperative PVO and early mortality, the use of sutureless technique in supracardiac or cardiac TAPVC was not completely promising (18, 20, 22) suggesting that the sutureless technique might not be beneficial for patients with unobstructed PVs. Although we aimed to explore the effectiveness of the sutureless repair for supracardiac or cardiac TAPVC, we did not identify significant differences between different subtypes of TAPVC (Table 3).

Compared with a previous meta-analysis (6), we observed a significant reduction in early mortality following the sutureless repair based on the latest evidence. Additionally, postoperative PVO and reoperations due to PVO following the sutureless repair were also alleviated. Furthermore, based on current evidence, we observed a lower early mortality with an OR of 0.40 favoring the sutureless technique. However, similar to previous findings, late mortality was still not significantly different between conventional surgery and the sutureless repair.

According to our inclusive criteria, we excluded several studies originating from the same institutions. Two studies (5, 25) from The Hospital for Sick Children of Toronto, one study (26) from the Guangdong Cardiovascular Institute, and one multicenter study (1) were excluded to avoid overlapping of data. Although the multicenter study performed by Shi et al. (1) had the largest TAPVC cohort, this study was excluded because it overlapped with the other two included studies (8, 19).

This updated meta-analysis explored the outcomes of primary sutureless repair, and compared the sutureless technique with conventional surgery for TAPVC. We acknowledged several limitations of this meta-analysis. First, all of the included studies were retrospective studies; consequently, the level of evidence was of low quality. Second, the baseline characteristics of our included two-arm studies (8, 18, 22) differed significantly concerning the TAPVC type, operative age and weight. Thus, the overall quality of this meta-analyses was compromised. Third, we only evaluated postoperative PVO, reoperations due to PVO, and early and late mortality. Outcomes such as postoperative ventilation and intensive care unit (ICU) stay were generally influenced by institutional protocols and perioperative management which were not necessarily associated with the effectiveness of surgical approaches. Therefore, we did not conduct a meta-analysis of these outcomes. Forth, we did not use rarely reported terms, such as TAPVC, TAPVD, or total anomalous pulmonary venous return, for the literature search. Therefore, using our search strategy might neglect some eligible studies. Finally, although SV and RAI were frequently observed in TAPVC, these covariates were not included in subgroup or meta-regression analysis because only three studies (15–17) reported pre-existing SV or RAI.

## Conclusion

In conclusion, following the sutureless technique, the incidences of postoperative PVO and reoperations due to PVO were 3.3 and 1.8%, respectively. Early and late mortality rates were 3.2 and 2.5%, respectively. Compared with conventional surgery, the sutureless technique significantly reduces postoperative PVO, reoperations due to PVO, and early mortality. However, there was no statistical difference between conventional surgery and the sutureless technique regarding late mortality. Prospective cohort studies or RCTs should be performed to assess the effectiveness of the sutureless technique for TAPVC repair.

## Data Availability Statement

The raw data supporting the conclusions of this article will be made available by the authors upon request.

## Ethics Statement

Ethical review and approval was not required for the study on human participants in accordance with the local legislation and institutional requirements. Written informed consent for participation was not required for this study in accordance with the national legislation and the institutional requirements.

## Author Contributions

LZ and YW conceived the study, designed the protocol, and collected the data. YW, ZP, and LS analyzed the data with input from CW. LZ and LS drafted the manuscript which was revised following critical review by all authors. All authors read and approved the final manuscript.

## Conflict of Interest

The authors declare that the research was conducted in the absence of any commercial or financial relationships that could be construed as a potential conflict of interest.

## Publisher’s Note

All claims expressed in this article are solely those of the authors and do not necessarily represent those of their affiliated organizations, or those of the publisher, the editors and the reviewers. Any product that may be evaluated in this article, or claim that may be made by its manufacturer, is not guaranteed or endorsed by the publisher.

## Supplementary Material

Supplementary Figure 1 Sensitivity analysis of incidence of postoperative PVO based on single-arm analysis. **(A)** Sensitivity analysis of incidence of postoperative PVO. **(B)** Sensitivity analysis of early mortality. **(C)** Sensitivity analysis of late mortality. **(D)** Sensitivity analysis of reoperations due to PVO.

Supplementary Figure 2 Sensitivity analysis of OR of postoperative PVO based on two-arm comparisons. **(A)** Sensitivity analysis of incidence of postoperative PVO. **(B)** Sensitivity analysis of early mortality. **(C)** Sensitivity analysis of late mortality. **(D)** Sensitivity analysis of reoperations due to PVO.
