# Supplementary material for: Sutureless Technique for Primary Total Anomalous Pulmonary Venous Connection Repair: An Updated Meta-Analysis
Source: Front Cardiovasc Med. 2022 Apr 28;9:890575. doi: 10.3389/fcvm.2022.890575 (PMC9095923; doi:10.3389/fcvm.2022.890575)
Supplement: Supplementary file 1 [file Table_1.DOCX]

**Table 1** Baseline characteristics of the included studies

| **First authors, year of publication, study area** | **Study design** | **Operative approaches** | **Study size**  **(n)** | **Category of TAPVC**  **(n)** | **Mean or median operative age**  **(days)** | **Preoperative PVO**  **(n)** | **Follow-up period**  **(months)** |
| --- | --- | --- | --- | --- | --- | --- | --- |
| Yoshimura, 2010, Japan^[14]^ | Case series | Sutureless  technique | 3 | Supracardiac 1  Infracardiac 2 | 12.7 | 3 | 15 |
| Azakie, 2011, USA^[15]^ | Case series | Sutureless  technique | 18 | Supracardiac 10  Infracardiac 5  Cardiac 1  Mixed 2 | 18 | 14 | 34 |
| Mueller, 2013, Switzerland^[16]^ | Case series | Sutureless technique | 7 | Supracardiac 4  Infracardiac 3 | 6.4 | 3 | 54 |
| Jung, 2016, Korea^[17]^ | Case series | Sutureless  technique | 21 | Supracardiac 9  Infracardiac 10  Mixed 2 | 21 | 13 | 3.6 |
| Lo Rito, 2015, Canada^[18]^ | Case-controlled studies | Sutureless  technique | 69 | Supracardiac 32  Infracardiac 16  Cardiac 6  Mixed 15 | 18 | 33 | 76.8 |
|  |  | Conventional surgery | 126 | Supracardiac 59  Infracardiac 20  Cardiac 33  Mixed 14 | 36 | 44 |  |
| Zhang, 2015, China^[19]^ | Case-controlled studies | Sutureless  technique | 70 | Supracardiac 57  Infracardiac 9  Mixed 4 | 68 | 19 | 12 |
|  |  | Conventional surgery | 70 | Supracardiac 58  Infracardiac 7  Mixed 5 | 55 | 19 |  |
| Zhu, 2019, China^[20]^ | Case-controlled studies | Sutureless  technique | 20 | Supracardiac | 198 | NA | 36 |
|  |  | Conventional surgery | 23 | Supracardiac | 202 |  |  |
| Shi, 2021, China^[21]^ | Case-controlled studies | Sutureless  technique | 15 | Infracardiac | 17 | 9 | 16 |
|  |  | Conventional surgery | 67 | Infracardiac | 27 | 36 | 35 |
| Liufu, 2021, China^[22]^ | Case-controlled studies | Sutureless  technique | 36 | Infracardiac | 21 | 21 | 12 |
|  |  | Conventional surgery | 27 | Infracardiac | 15 | 13 |  |
| Qiu, 2021, China^[23]^ | Case-controlled studies | Sutureless  technique | 43 | Supracardiac | 60 | 13 | 65 |
|  |  | Conventional surgery | 130 | Supracardiac | 90 | 39 |  |
| Xia, 2021, China^[24]^ | Case-controlled studies | Sutureless  technique | 13 | NA | 90 | 13 | 85 |
|  |  | Conventional surgery | 13 |  | 90 | 13 |  |

*TAPVC* Total anomalous pulmonary venous connection, *NA* Not available
