# Supplementary figures and images for: Sutureless Technique for Primary Total Anomalous Pulmonary Venous Connection Repair: An Updated Meta-Analysis
Source: Front Cardiovasc Med. 2022 Apr 28;9:890575. doi: 10.3389/fcvm.2022.890575 (PMC9095923; doi:10.3389/fcvm.2022.890575)

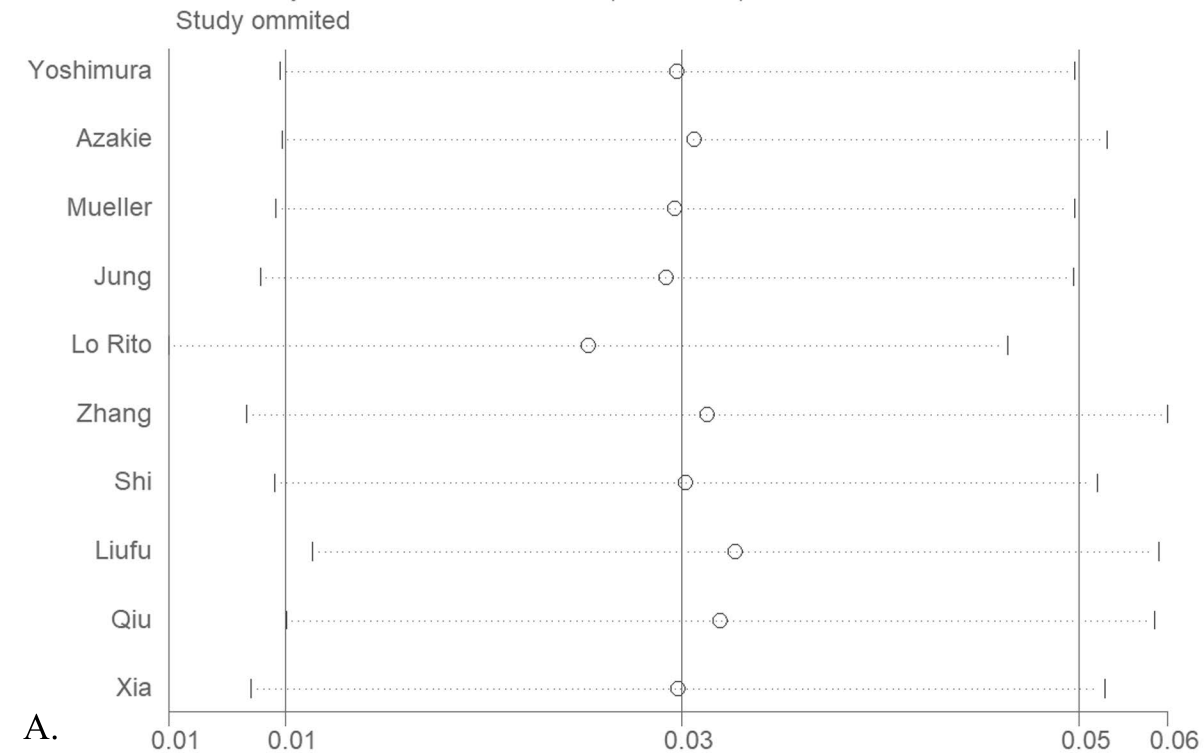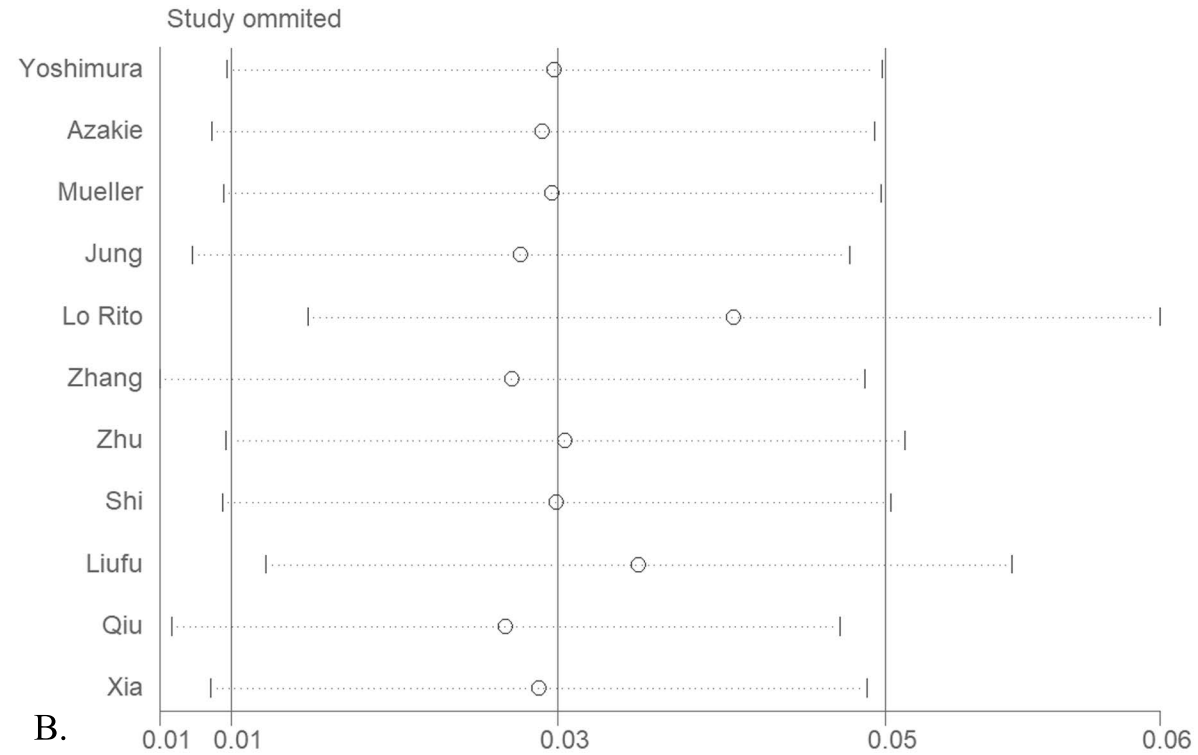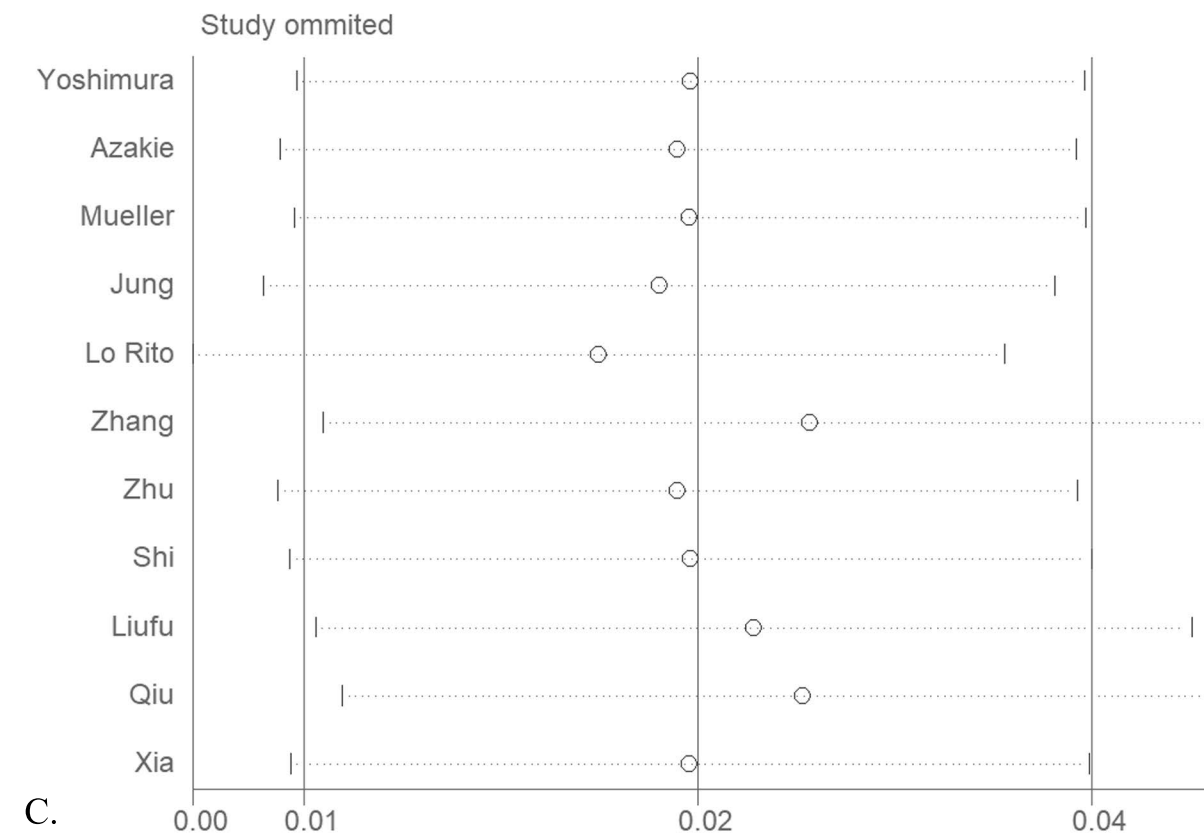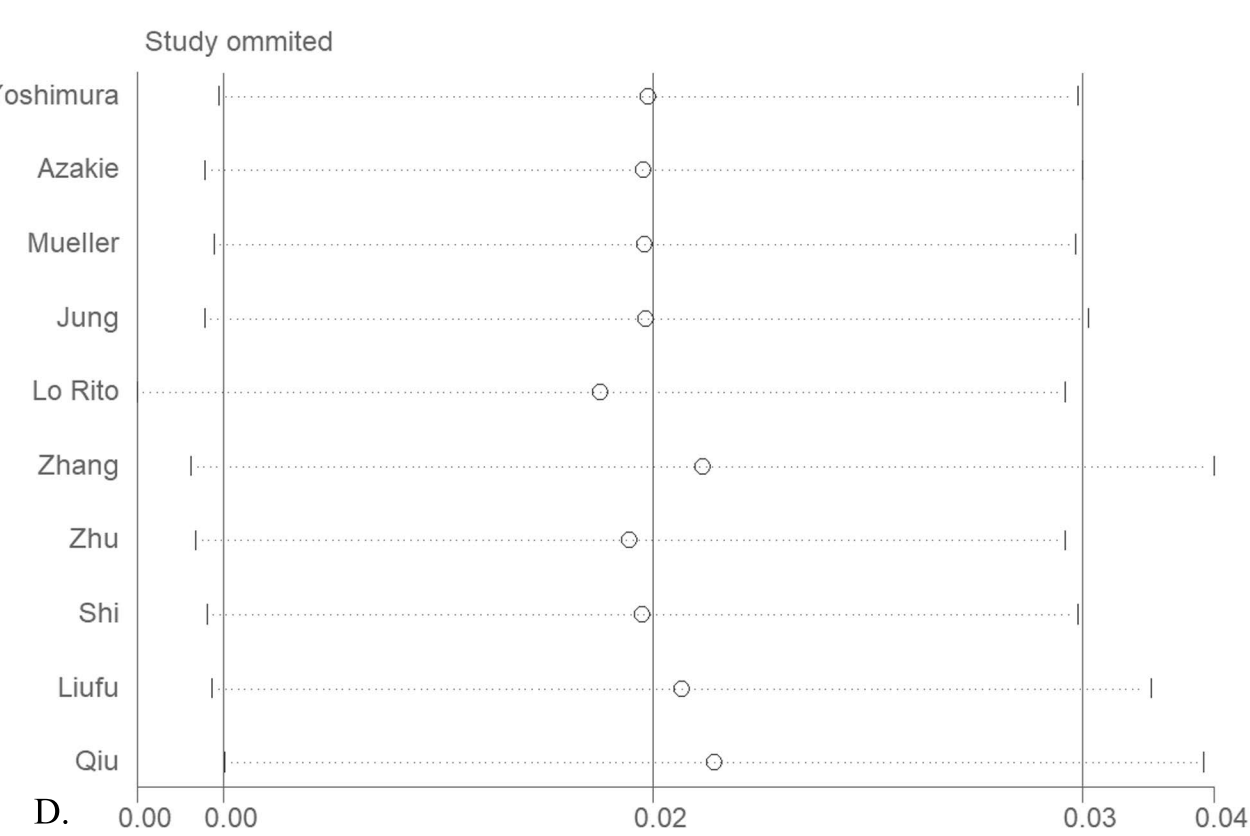

Supplement: Supplementary Figure 1 — Sensitivity analysis of incidence of postoperative PVO based on single-arm analysis. (A) Sensitivity analysis of incidence of postoperative PVO. (B) Sensitivity analysis of early mortality. (C) Sensitivity analysis of late mortality. (D) Sensitivity analysis of reoperations due to PVO. [file Data_Sheet_1.PDF]

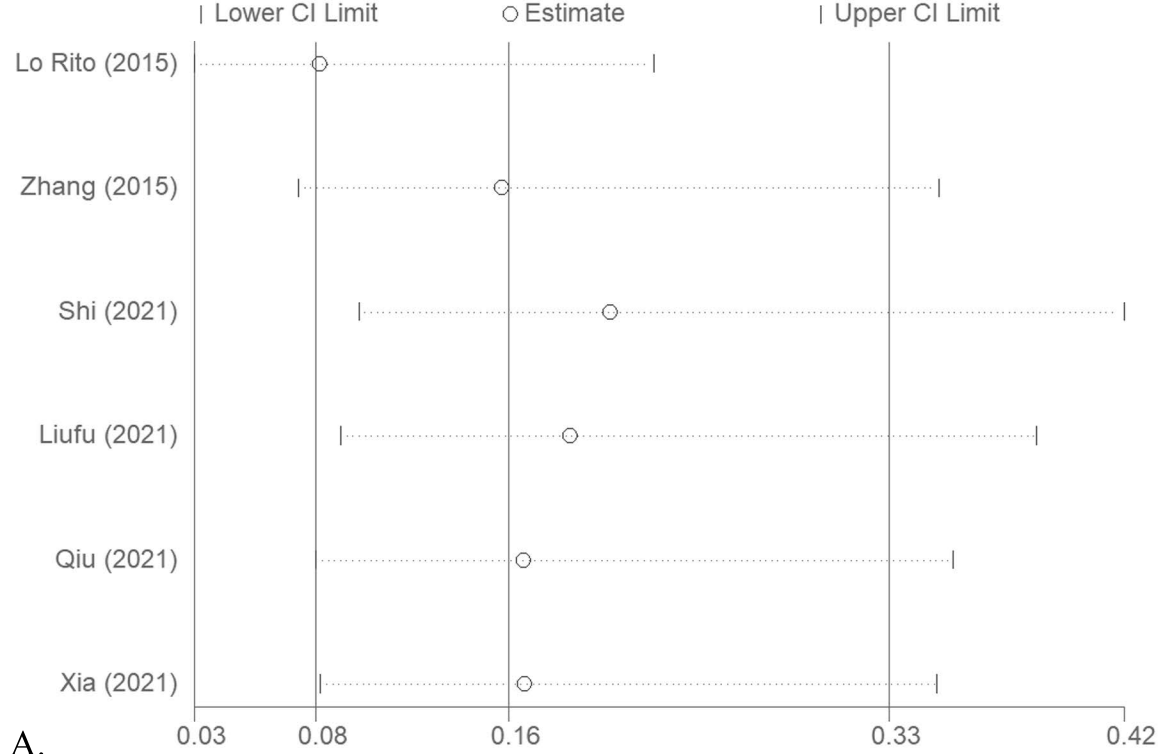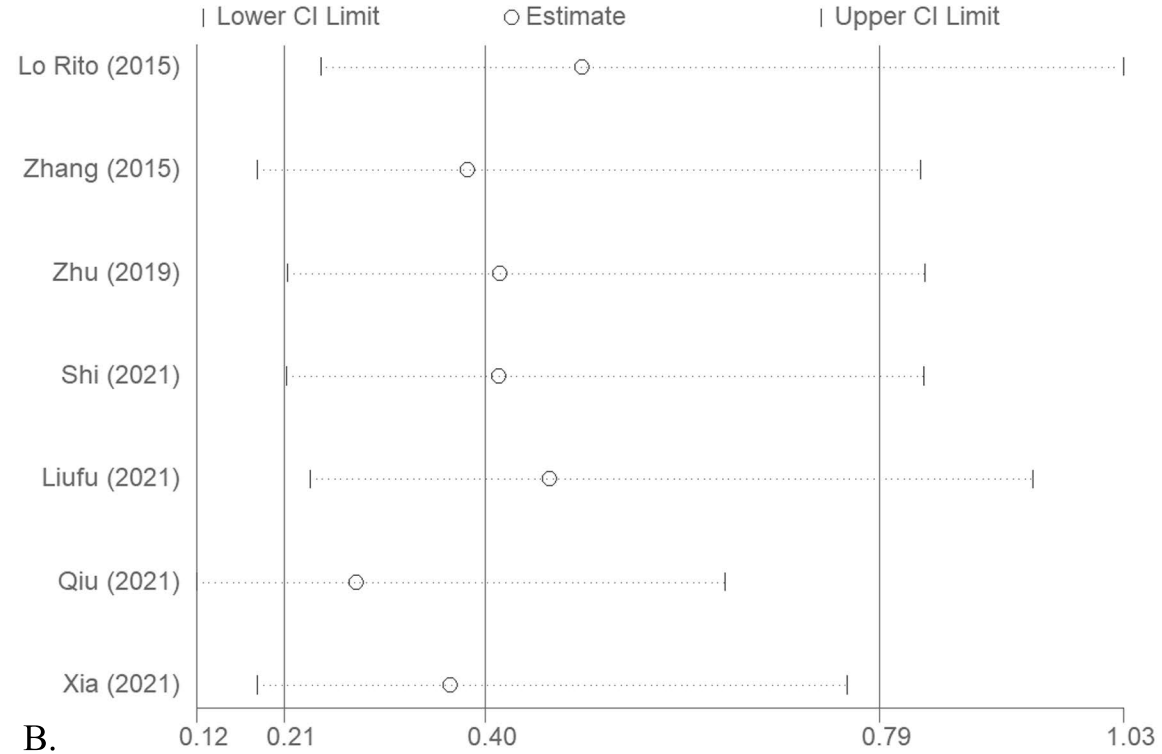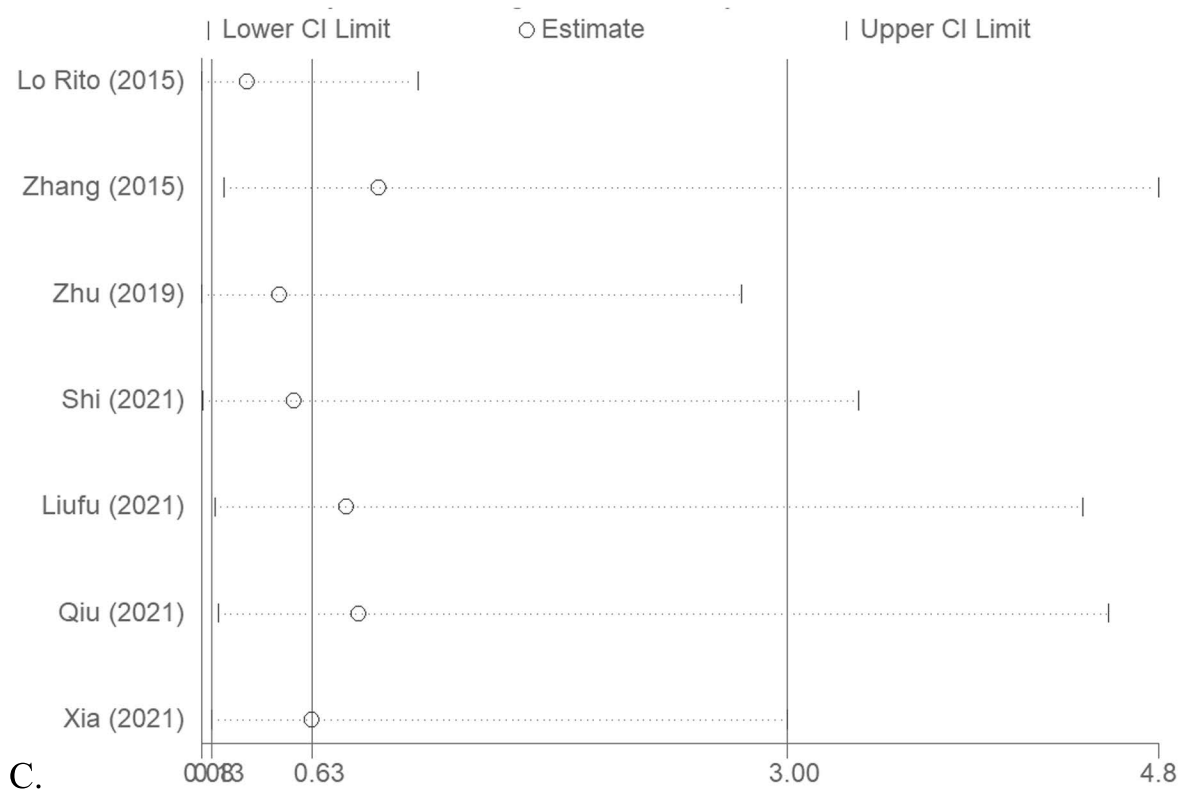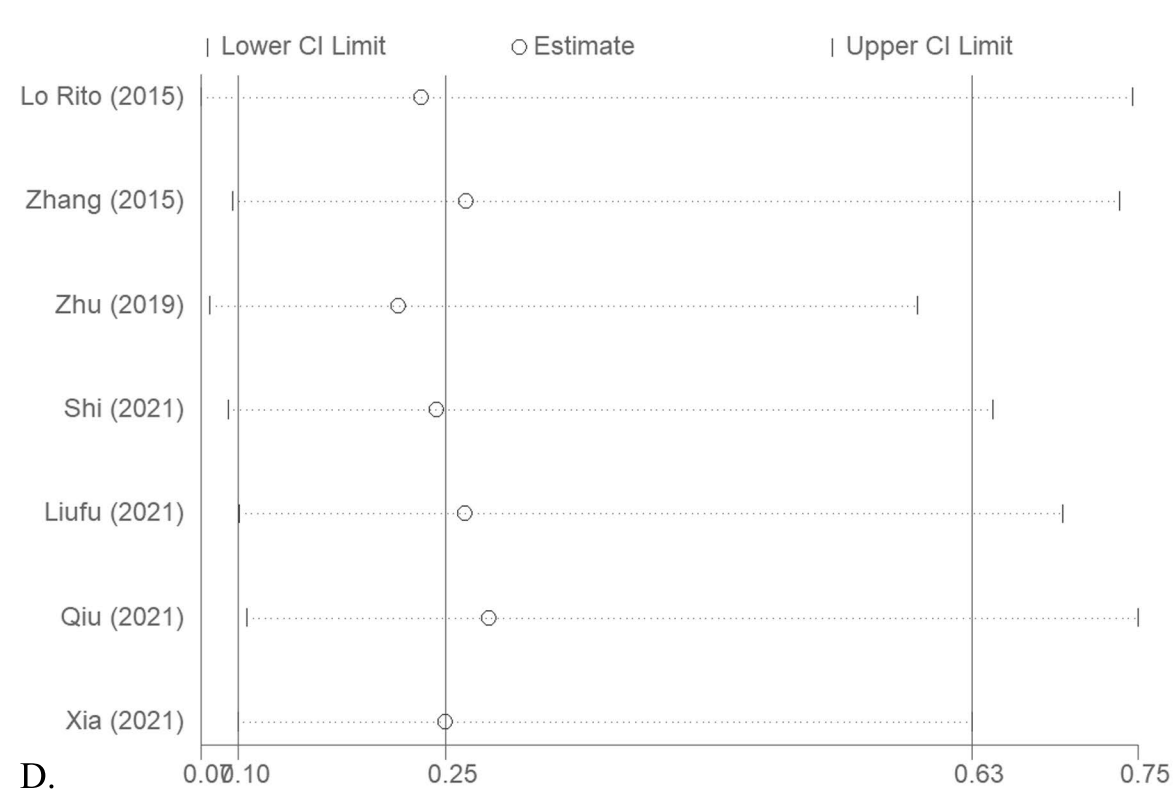

Supplement: Supplementary Figure 2 — Sensitivity analysis of OR of postoperative PVO based on two-arm comparisons. (A) Sensitivity analysis of incidence of postoperative PVO. (B) Sensitivity analysis of early mortality. (C) Sensitivity analysis of late mortality. (D) Sensitivity analysis of reoperations due to PVO. [file Data_Sheet_2.PDF]
